# Geospatial modelling of lymphatic filariasis and malaria co-endemicity in Nigeria

**DOI:** 10.1093/inthealth/ihad029

**Published:** 2023-04-24

**Authors:** Obiora A Eneanya, Lisa J Reimer, Peter U Fischer, Gary J Weil

**Affiliations:** Infectious Diseases Division, Department of Medicine, Washington University School of Medicine, St. Louis, MO 63108, USA; Department of Vector Biology, Liverpool School of Tropical Medicine, Liverpool L3 5QA, UK; Infectious Diseases Division, Department of Medicine, Washington University School of Medicine, St. Louis, MO 63108, USA; Infectious Diseases Division, Department of Medicine, Washington University School of Medicine, St. Louis, MO 63108, USA

**Keywords:** lymphatic filariasis, malaria, Nigeria

## Abstract

**Background:**

Lymphatic filariasis (LF) and malaria are important vector-borne diseases that are co-endemic throughout Nigeria. These infections are transmitted by the same mosquito vector species in Nigeria and their transmission is similarly influenced by climate and sociodemographic factors. The goal of this study was to assess the relationship between the geospatial distribution of both infections in Nigeria to better coordinate interventions.

**Methods:**

We used national survey data for malaria from the Demographic and Health Survey dataset and site-level LF mapping data from the Nigeria Lymphatic Filariasis Control Programme together with a suite of predictive climate and sociodemographic factors to build geospatial machine learning models. These models were then used to produce continuous gridded maps of both infections throughout Nigeria.

**Results:**

The R^2^ values for the LF and malaria models were 0.68 and 0.59, respectively. Also, the correlation between pairs of observed and predicted values for LF and malaria models were 0.69 (95% confidence interval [CI] 0.61 to 0.79; p<0.001) and 0.61 (95% CI 0.52 to 0.71; p<0.001), respectively. However, we observed a very weak positive correlation between overall overlap of LF and malaria distribution in Nigeria.

**Conclusions:**

The reasons for this counterintuitive relationship are unclear. Differences in transmission dynamics of these parasites and vector competence may contribute to differences in the distribution of these co-endemic diseases.

## Introduction

Lymphatic filariasis (LF), caused by the parasitic nematode *Wuchereria bancrofti*, and malaria (mostly caused by the protozoan *Plasmodium falciparum*) are important vector-borne diseases that are responsible for large public health burdens in sub-Saharan Africa (SSA).^1,2^ LF and malaria are co-endemic throughout Nigeria,^3,4^ where they are predominantly transmitted by *Anopheles* mosquitoes.^5^ Infection with *W. bancrofti* is often asymptomatic, although infected individuals may manifest with lymphoedema, hydrocele and acute adenolymphangitis attacks.^6^ Falciparum malaria is a leading cause of mortality in SSA and this is especially true for children <5 y of age and pregnant women.^7^

Although LF and malaria account for considerable morbidity and mortality in regions where both infections are present, relatively little is known regarding the interaction of these parasites in human populations. A prior study on this subject found a counterintuitive negative spatial correlation between these infections in the four adjacent countries: Ghana, Burkina Faso, Togo and Benin in West Africa.^8^ However, their study was based solely on previously published falciparum malaria prevalence maps^9^ that were then used to assess the degree of spatial correlation with bancroftian filariasis. The LF and malaria maps were constructed using different modelling approaches as well as different sets of environmental covariates.^9,10^ Maps produced using the same modelling methods and the same set of covariates are ideal in order to maintain consistency in model predictions.

A mathematical modelling study found a similar negative association and suggested that attempts to control one of these parasites may inadvertently result in increased susceptibility to infection to the other in the mosquito vector and in humans.^11^ Although these findings are in keeping with previous work,^8^ Slater et al.,^11^ in their co-infection model, included an additional mortality parameter in filarial-infected mosquitoes that was double the estimated daily death rate. Such assumptions may yield model results that show a negative correlation between LF and malaria due to the dependence of vectorial capacity on daily survival. In general, the iterative process of fitting simulation models whereby different values of model parameters are tested in an attempt to account for a wide range of possible biological scenarios makes it more difficult to interpret model outputs. It is therefore necessary to test this hypothesis with representative data from population field studies.

In order to assess whether the spatial correlation between LF and malaria can also be found in Nigeria, we built on prior work on the geospatial distribution of LF in Nigeria.^3^ In this study we used nationally representative prevalence data for both LF and malaria and a suite of explanatory environmental and sociodemographic covariates to train a machine learning model. The trained model was then used to produce predictive prevalence maps for locations that did not have survey data. We then used spatial data to assess the degree of correlation for the distribution of LF and falciparum malaria in Nigeria.

## Methods

### LF and malaria prevalence data

**Figure 1.**
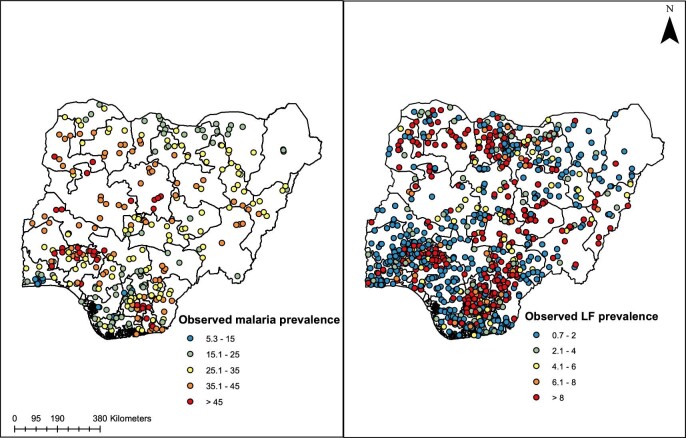
Location of study sites showing the prevalence of malaria (left) and LF (right) surveys in Nigeria.

LF data were from pre-intervention site-level parasitological surveys conducted by the Nigerian Ministry of Health during the national mapping of LF from 2000 to 2013.^12^ This is a nationally representative dataset of baseline LF prevalence for Nigeria (Figure 1). A full description of these data were provided in our previous studies^3,13^ and in the Neglected Tropical Disease Master Plan of the Nigerian Ministry of Health.^12^ Briefly, states in Nigeria are subdivided into local government areas (LGAs), which represent the third administrative unit of government. Surveys were conducted to ensure that all LGAs had at least one survey location, although more than one location was surveyed in larger LGAs. Locations for survey sites were selected based on distances between them; all survey sites were at least 50 km apart.^12^ At each site, the survey aimed to enrol between 50 and 100 adults (age >15 y). Testing for the presence of filarial antigenemia in finger prick blood was done using rapid immunochromatographic card tests (ICTs; Alere, Scarborough, ME, USA). This modelling exercise used 1327 survey data points with ICT results from approximately 143 000 survey participants.

Our study used malaria prevalence data collated from the 2015 Nigerian Malaria Indicator Survey (NMIS).^14^ The survey was jointly implemented by the National Malaria Elimination Programme, National Population Commission and the National Bureau of Statistics with technical support provided by ICF International under the Demographic and Health Surveys Programme of the United States Agency for International Development. The main objectives of the NMIS were to provide information on key malaria indicators such as prevalence, treatment and prevention practices and to determine the dominant *Plasmodium* species that were circulating within the population.

The primary sampling frame for the NMIS was the cluster, which is a collection of households within an enumeration area (EA). These EAs were used for national censuses and are comprised of communities within an LGA. For the 2015 NMIS, 25 households were selected in each cluster by equal probability systematic sampling. All children 6–59 months old within a household were eligible for inclusion. Finger (or heel) prick blood samples were collected for detection of malaria parasitaemia by microscopy. The dataset used for this modelling exercise contained a total of 333 clusters (138 clusters in urban areas and 195 clusters in rural areas) and they included data from >8000 households. A complete description of the survey design and testing procedures can be found in the 2015 NMIS report (https://dhsprogram.com/pubs/pdf/MIS20/MIS20.pdf).^14^

### Environmental and sociodemographic data

A suite of environmental and sociodemographic covariates were obtained from open access sources. All covariates considered for this work had biologically plausible links to both LF and malaria. Continuous gridded maps of altitude, rainfall and temperature were processed from the WorldClim database (https://www.worldclim.org/), which provides long-term (>50 y) averages of data collated from weather stations distributed across the world. Data on *Anopheles* spp. distribution were downloaded from the Malaria Atlas Project (https://malariaatlas.org/). This resource curates malariometric data that includes factors that influence malaria transmission and intervention coverages. Finally, data on healthcare access and poverty were downloaded from the WorldPop database (https://www.worldpop.org/), which provides modelled estimates of various developmental and demographic indicators. Incorporating potential disease drivers within a geostatistical framework is known to improve model predictions. Also, in addition to these explanatory covariates, we included the geographical coordinates of the observed data to account for the effects of spatial heterogeneity of the survey locations. This practice is known to further improve model predictions when machine learning models are used for spatial analysis.^15^

All input grids were resampled to a common spatial resolution of 5 km×5 km using the nearest neighbour algorithm.^16^ The stack of raster files were aligned and clipped to the geographical extent of mainland Nigeria.

### Geospatial analysis

We used the quantile regression forest (QRF) model to produce smooth prevalence maps of LF and malaria for areas without ground-truth data.^17^ QRF is an ensemble machine learning algorithm for classification and regression that handles complex and multidimensional data well. It has been demonstrated to outperform traditional regression models under comparable modelling scenarios.^18^ A complete description of this modelling framework can be found in our previous publications.^3,19^

For this analysis, we first implemented a random forest (RF) model. The RF model is used to tune parameters for use in the QRF model. This process informs the optimum number of covariates to be considered for each recursive node split in the QRF model. For each directly modelled response variable (in this case, LF and malaria), we trained the model on a random subset of 70% of the data points, while the remaining 30% were used for model validation. We performed a 10-fold cross-validation on out-of-bag samples, repeating this process five times.

For model performance metrics, we computed the root mean squared error (RMSE), R^2^ and Pearson's coefficient between pairs of observed vs predicted values for both the LF and malaria models. We used a two-step process to compute the association between LF and malaria prevalence. We first used observed prevalence data for LF for every location with ground-truth data and then extracted corresponding values from raster layers of the modelled predictions for malaria. Then we performed this analysis in reverse by using ground-truth prevalence data for malaria to extract corresponding values for LF from the predicted model output for LF. We then used these two data sets to compute Pearson's coefficients to determine the relationship between paired observed vs predicted values for LF and malaria. In addition, using our ground-truth LF prevalence data, we extracted corresponding values for malaria prevalence using previously modelled malaria prevalence maps for the year 2000 and 2005.^20^ The purpose of this extra analysis was to test our model on malaria data prior to large-scale uptake of malaria interventions such as bed net usage. This rules out any temporal effects that may have occurred from malaria interventions.

All analyses were done in R (version 4.1.3; R Foundation for Statistical Computing, Vienna, Austria).^21^ The raster package was used for processing and preparing of environmental covariates. RF and QRF models were implemented using the randomForest and quantregForest packages, respectively. Output raster maps of final model predictions were projected at a spatial resolution of 5 km×5 km. These raster layers were then imported into ArcGIS (version 10.6.1; Esri, Redlands, CA, USA) for visualization.

## Results

### Model performance indicators

Table 1 shows the performance indicators for the trained QRF models for LF and malaria. Here, R^2^ values were 68% and 59% for LF and malaria, respectively. This indicates that the covariates used in model fitting were able to explain the majority of the variability in model predictions. Highly significant Pearson's coefficients between observed and predicted values for LF (0.69 [95% confidence interval {CI} 0.61 to 0.79], p<0.001) and malaria (0.61 [95% CI 0.52 to 0.71], p<0.001) also indicate the validity of these models.

**Table 1.** Model performance indicators

Disease	RMSE	R^2^	Correlation between pairs of observed and predicted values (95% CI)	p-Value
Lymphatic filariasis	1.18	0.68	0.69 (0.61 to 0.79)	<0.001
Malaria	1.23	0.59	0.61 (0.52 to 0.71)	<0.001

### Variable importance plot

**Figure 2.**
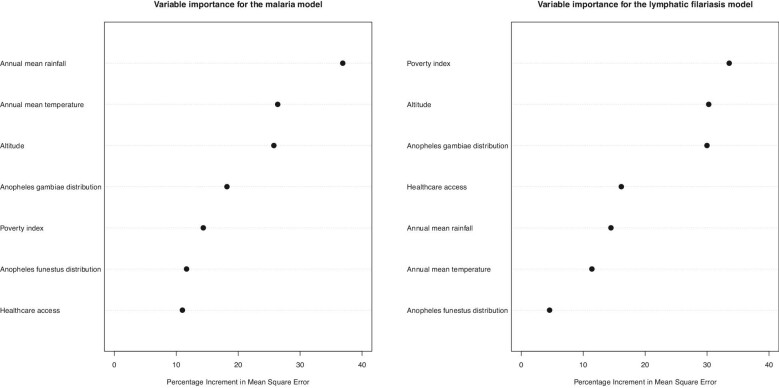
Variable importance for the trained malaria and LF models.

Figure 2 shows the variable importance plot of the QRF model trained using LF and malaria prevalence data. Here, the percentage increment in mean squared error shows that the climatic variables rainfall, temperature and altitude as well as *Anopheles gambiae* distribution were the most informative variables in the malaria model. For the LF model, sociodemographic variables such as poverty index and healthcare access as well as altitude and *A. gambiae* distribution were important predictors.

### Predicted LF and malaria prevalence

**Figure 3.**
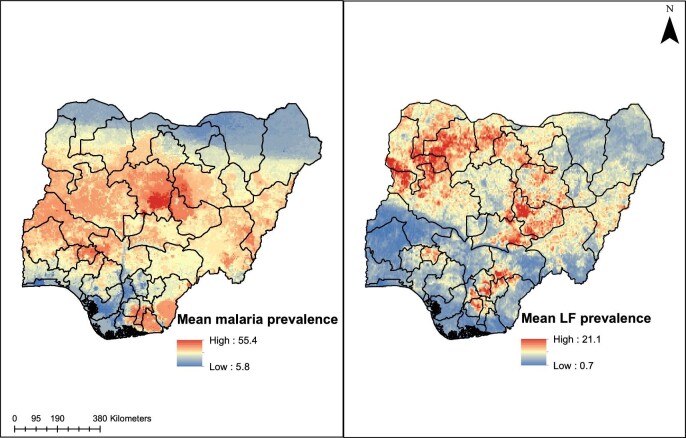
Mean predicted maps of endemicity of malaria and LF for Nigeria at 5 km×5 km spatial resolution.

The 5 km×5 km predicted maps of LF and malaria for Nigeria are presented in Figure 3. As can be seen, the endemicity patterns in these maps are largely dissimilar. Importantly, there was a very weak positive correlation in the association between LF and malaria; the Pearson's coefficient computed using pairs of data from observed prevalence values for LF and corresponding values extracted from predicted prevalence of malaria was 0.08 (95% CI 0.02 to 0.13; p=0.006). When this analysis was computed in reverse, i.e. using observed malaria prevalence and extracting corresponding values from predicted LF prevalence, the Pearson's coefficient was 0.062 (95% CI 0.03 to 0.12; p=0.006). Furthermore, when we performed this analysis using previously modelled malaria prevalence for Nigeria for 2000 and 2005,^20^ the Pearson's coefficient was −0.01 (95% CI −0.06 to 0.04; p=0.71) and −0.0006 (95% CI −0.05 to 0.05; p=0.98) for 2000 and 2005, respectively.

**Figure 4.**
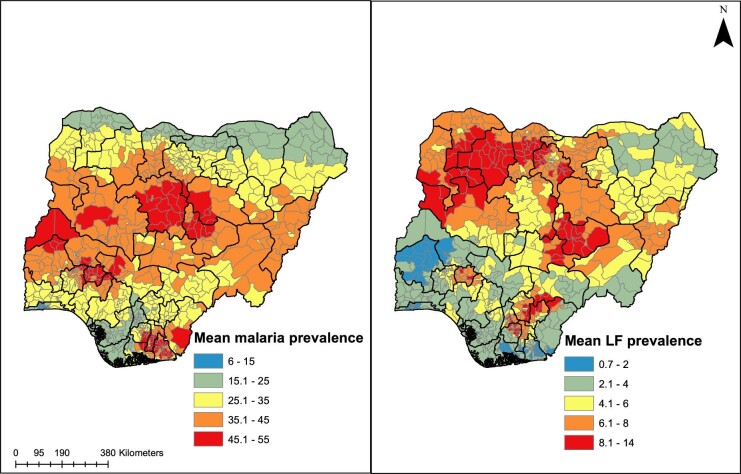
District-level mean predicted prevalence maps of malaria and LF.

As districts (i.e. LGAs) constitute the primary administrative division for mass drug administration (MDA) programs for LF and bed net distribution and other interventions against malaria, we have reprojected these maps to delineate district-level estimates. Maps presented in Figure 4 are the district-level mean predicted prevalence of malaria and LF in Nigeria. States in the northwestern, northcentral (especially Plateau and Nasarawa states) and pockets in the southeastern region appear to be highly endemic for LF. In contrast, the malaria endemicity map indicates high or moderate endemicity in most areas of Nigeria apart from the northern-most regions.

## Discussion

This study has used extensive nationally representative datasets for bancroftian filariasis and falciparum malaria to examine correlations and the extent of geographical overlap of these two vector-borne infections in Nigeria. Our results show that the distribution of these infections is only weakly correlated despite the fact that they are both transmitted by anopheline vectors. Indeed, some areas with the highest LF prevalence had little malaria, and vice versa. This counterintuitive result has been previously reported.^8,11,22,23^ Confirmatory studies from other countries are extremely important to elucidate whether the lack of positive association between the two *Anopheles* transmitted parasites, *W. bancrofti* and *P. falciparum*, is a local phenomenon or a common rule in larger parts of Africa.

Previous studies suggest that a possible explanation for the weak relationship between LF and malaria may be related to historical vector control interventions.^8^ Nigeria had received extensive bed net coverage and indoor residual spraying (albeit to a lesser extent) for many years. Taken together, these interventions may well have been sufficient to reduce the transmission of LF to a greater extent than for malaria due to the inefficiency of transmission of the filarial parasite.^24^ Given the mismatch in dates of LF and malaria surveys that were used in this work (i.e. LF data from 2000 to 2013 and malaria data for 2015), we further tested this hypothesis by applying our models on malaria prevalence for 2000 and 2005. This was prior to extensive scale-up of malaria intervention campaigns. Using previously modelled maps from Bhatt et al.,^20^ we found no significant correlation between LF and malaria. It is also possible that the interplay between the transmission of both infections at a population level may be influenced by years of MDA with ivermectin for onchocerciasis control. Although ivermectin is primarily used for the treatment of filarial worm infections, studies have found that it reduces both the survival and density of *Anopheles* mosquitoes.^25–27^

Also, seasonality, specific climate and sociodemographic factors may explain the mismatch in transmission patterns. Our analysis on the variable importance for the LF and malaria models indicate that climatic variables like rainfall and temperature had the greatest influence on the distribution of malaria in Nigeria. This is distinctively shown in the maps presented in Figures 3 and 4, where malaria is seen to be less prevalent in the dryer northernmost parts closer to the Sahara desert regions of Nigeria and in rainy southern rainforest regions. There is evidence that mosquito populations do not thrive in areas with heavy rainfall, as this tends to wash out breeding sites.^28^ Conversely, sociodemographic factors such as the poverty index and healthcare access were important correlates for filariasis distribution. This is interesting and suggests that perhaps LF infection is more common in poor rural settings without access to adequate healthcare infrastructure and less related to climate than malaria.

The different transmission dynamics of both parasites could lead to different relationships between biting density and disease incidence. Unlike malaria, filarial parasites do not replicate within the vector and the number of infective larvae inoculated by one mosquito will not lead to infection. It has been estimated that thousands of infective bites are required for a patent LF infection,^29^ whereas a single inoculation with sporozoites can cause malaria. Therefore the intensity of exposure required to sustain community transmission is lower for malaria than LF. In addition, vector competence to each parasite and overall vectorial capacity are species specific.

Although it is known that *A. gambiae* is the dominant mosquito vector for both LF and malaria in SSA,^2,5^ concurrent transmission of LF and malaria parasites by a single vector is rare in nature.^30^ Furthermore, there are several subspecies within the *A. gambiae* complex (*A. gambiae* s.l). Souza et al.^31^ highlight that these anophelines differ in terms of distribution, host preference, transmission potential, resistance status and immunity. For instance, they report that *Anopheles melas* is a more competent LF vector because it has a less extensive cibarial armature that damages microfilariae on ingestion.^31^ Extensive diversity among anophelines within a given geographical region may differentially influence transmission of LF and malaria. In addition, it is thought that infection with *W. bancrofti* increases the susceptibility of mosquitoes to *P. falciparum* infection, because migration of microfilariae can disrupt the peritrophic midgut membrane and facilitate *Plasmodium* invasion.^32^

The World Health Organization has recommended integrated vector management (IVM) for the control of LF and malaria in co-endemic areas and areas where both diseases share mosquito vectors.^33^ We and others agree with that recommendation.^34–36^ However, this can only be a supplemental intervention strategy for LF elimination and malaria control. Targeted deployment of IVM may be more effective if it is informed by district-level data on vector ecology, on the epidemiology of both diseases and on compliance with MDA with ivermectin plus albendazole for LF elimination.

Our findings further demonstrate a very weak correlation between the spatial distributions of malaria and LF in Nigeria. A stronger correlation would have increased the importance of large-scale integration of control activities and surveillance for these diseases. Although we have suggested several possible explanations for this counterintuitive result, we cannot fully explain it at this time. Field studies specifically designed to test for the presence of LF and malaria parasites within the same human and vector populations may shed more light on interactions between these parasites.

## Data Availability

The DHS Programme is a repository body for DHS datasets. Although access to this dataset is not freely available to the public, the DHS Programme can provide access to the datasets after formal application and registration.
